# Systemic Therapy of Psoriasis in Children—Proposal of an Algorithm for Interdisciplinary Teamwork

**DOI:** 10.3390/jcm13216307

**Published:** 2024-10-22

**Authors:** Nesrine Ben-Anaya, Matthias Augustin, Fabian Speth, Roman Scheidmann, Brigitte Stephan

**Affiliations:** 1 Institute for Health Services Research in Dermatology and Nursing (IVDP), University Medical Center Hamburg-Eppendorf (UKE), 20246 Hamburg, Germany; n.ben-anaya@uke.de (N.B.-A.); m.augustin@uke.de (M.A.); 2 Clinic for Child and Adolescent Medicine (Kinder-UKE), University Medical Center Hamburg-Eppendorf (UKE), 20246 Hamburg, Germany; f.speth@uke.de; 3 Pediatrics, Pediatric Pulmonology, Altona Children’s Hospital of Hamburg (AKK), 22763 Hamburg, Germany; roman.scheidmann@kinderkrankenhaus.net

**Keywords:** interdisciplinary, children, psoriasis

## Abstract

**Background/Objectives:** Children and adolescents with psoriasis can have severe and long-lasting disease requiring early and effective therapy. The range of associated comorbidities is comparable to adult patients with additional problems deriving from their growth and maturation. Therefore, tailored information and interdisciplinary teamwork is necessary to effectively manage pediatric psoriasis. **Methods:** We reflected on our experience with therapy management of children and adolescents with psoriasis coming to our university outpatient clinic and summarized the challenges and special features of these patients together with approved medications and recommendations for treatment. We present our algorithm for managing these patients in an interdisciplinary setting. **Results:** Children can develop psoriasis very early in their life, and they show specific patterns of skin involvement depending on age. Scores such as the cDLQI and the PASI help to quantify the clinical severity and burden of the disease, and the upgraded criteria should reflect that children’s needs are different from adults’. The choice of medication is limited to a few, but increasing approvals for children and the close exchange of information and preparations with pediatricians and other specialties before initiating systemic therapies are crucial for children to support compliance. We emphasize the focus on vaccinations and the treatment of chronic infections, e.g., the management of TBI, which is different from adults. **Conclusions:** With the increased options for the systemic treatment of children with psoriasis, clear and adapted information for the child, guardian and pediatrician is essential to assure a well-managed environment and to prevent the unnecessary termination of effective therapy.

## 1. Introduction

Psoriasis is a common, chronic immune-mediated systemic disease with a prevalence of 2.0–3.5% of the general population [1,2]. It is not limited to the skin but also affects the joints in about 20% of all cases, and associated comorbidities include metabolic syndrome, depression, other autoimmune diseases such as chronic inflammatory bowel syndrome, uveitis or osteitis, and chronic recurrent multifocal osteomyelitis (CRMO).

Pediatric psoriasis is not uncommon, and the prevalence rates vary according to age, sex, geographical location, study design, and case definition [3]; furthermore, pediatric psoriasis is also associated with systemic disorders, such as anemia, failure to thrive, and colitis.

Children and adolescents with psoriasis can have severe and long-lasting disease requiring early and effective therapy. As a majority of (systemic) treatments are not approved for use in children, the treatment of pediatric psoriasis remains a challenge. With new studies, the options for effective treatments are expanded, and more pediatricians send their young patients for dermatology consultation. We realize that there is an increasing need for information because it differs from adults seeking therapy, as well as a need for close interdisciplinary teamwork for successful guidance through a treatment regimen.

## 2. Materials and Methods

In the Institute for Health Services Research in Dermatology and Nursing (IVDP) of the University Medical Center Hamburg-Eppendorf (UKE), Hamburg, Germany, we run a specialized outpatient clinic for chronic inflammatory skin diseases, and we regularly see children with moderate to severe psoriasis who need systemic therapy. Therefore, we realized the importance of addressing pediatric issues in disease management and that therapy needs to be conducted differently from our routine with adults. This leads to close interdisciplinary coworking and the exchange of knowledge and information between medical specialties caring for children with this disease. We present here the basis for our algorithm in coordinating the therapy of children with psoriasis, which proved to be practical and effective in our daily work with children and their guardians in managing pediatric psoriasis.

## 3. Results

### 3.1. Epidemiology of Pediatric Psoriasis

Psoriasis can occur at any age. In about one-third of those affected, it begins in the first two decades of life. Its prevalence varies considerably in different parts of the world and is particularly high in Europe. The prevalence for children and adolescents under the age of 18 in Germany is estimated to be about 0.71% and shows an almost linear increase from 0.13% in 0- to 2-year-olds to 0.67% in 14- to 18-year-olds in Germany [4]. In the USA, the annual incidence of children diagnosed with psoriasis increased (doubled) within 30 years, which might be due to the increase in certain risk factors such as psychosocial stress, infections, and obesity [5,6]. In contrast, prevalence data are considerably lower in China with 0.05% [1].

While the established paradigm posits bacterial infections as the predominant factor influencing this process, there is evidence indicating an escalating involvement of viral and fungal infections in the underlying pathophysiology of psoriasis. The theory pertaining to super antigen engagement holds relevance in the context of both bacterial and viral infections [6].

With early onset, a positive family history is often found as an indication of a strong genetic component. Frequently, the disease continues into adulthood with the same type of psoriasis. However, a premature onset of plaque psoriasis does not seem to be associated with greater severity of the disease [7].

The interplay between psoriasis and hormonal fluctuations across different life stages has been documented; however, it remains to be conclusively characterized. Notably, in the context of females, hormonal shifts such as those transpiring during puberty have been implicated in the initiation or exacerbation of psoriasis. Further studies underscore the association between psoriasis onset and the pubertal phase, as a substantial number of individuals tend to experience their inaugural psoriatic lesions shortly after puberty, coinciding with hormonal level reductions. A contemporary investigation highlighted how hormonal fluctuations during the menstrual cycle can impact psoriasis dynamics [8,9].

### 3.2. Special Features of Juvenile Psoriasis

In infancy and early childhood, psoriasis often manifests in the diaper region, with possible misdiagnosis as fungal diaper rash, and later onset usually first occurring in areas including the scalp, extremities, and trunk [10], and might be misdiagnosed as fungal diaper rash.

According to a study of 887 patients < 18 years of age, the most common subtypes of psoriasis are as follows: plaque psoriasis (73.7%); followed by guttate psoriasis (13.7%) often after upper respiratory tract infections, e.g., with streptococci; scalp psoriasis (7.6%); and pustular psoriasis (1.1%) [5]. Significant variations in nail psoriasis prevalence are worth mentioning, spanning from 0.6% to as high as 79% among infants [11]. One of the main impacts on quality of life of children is severe itching of the skin lesions with a significant influence on school performance and social development [12,13].

### 3.3. How Can We Measure the Severity of the Disease?

The PASl (Psoriasis Area and Severity Index), BSA (Body Surface Area), and PGA (Physician’s Global Assessment) are most commonly used to quantify the clinical severity of plaque psoriasis [14], and the Children’s Dermatology Life Quality Index (CDLQI) is used to assess the burden of the disease.

The calculation of the PASI includes the percentage of areas affected, as well as the severity of erythema, plaque thickness, and scaling, which are assessed for the four sites of the head, arms, trunk (up to the groin), and legs (including buttocks). The total score ranges from 0 (no psoriasis) to 72 (most severe clinical picture). A score of 10 or more indicates moderate to severe disease, which is considered an indication for initiating systemic therapy. It should be noted that the PASI has not been validated for use in children and that infestation of the nails is not taken into account. In addition, the severity of the erythema is underestimated in dark-skinned individuals [14]. The PASI correlates only moderately with the CDLQI. With the BSA, the severity of psoriasis is defined only by the affected body surface area. A moderate to severe psoriasis affects at least 10% of the body surface area. The PGA, with gradations from 0 to 4, is used as a rough measure of severity including erythema, infiltration, and scaling, but not the extent [14].

According to a study by Manzoni et al. [15], psoriasis was one of the skin diseases that had the most negative influence on one’s quality of life (QOL). Using the Infant Dermatitis Quality of Life Index score (IDQoL), a regression analysis was performed, which concluded that patients with psoriasis had 2.7 times more impaired QOL as compared to the general pediatric population. Compared to atopic dermatitis, psoriasis had a higher impact on patients within the following categories: having a symptomatic cutaneous state and suffering from sleep disturbance secondary to their underlying disease.

Questions that promote conversations about the effects of psoriasis on the patient’s home, school, and social lives are an important part of the overall management. Health care providers may consider using a formal QOL instrument as part of the overall evaluation and rationale for using systemic therapies, especially in the setting of worsening disease. The most commonly used QOL screen for pediatric psoriasis is the Children’s Dermatology Life Quality Index. Parents should be aware of these relevant factors and the need for effective therapy to support compliance, as well as awareness for comorbidities and the need for therapy regarding those as well.

In addition, we recommend, as with adults, the application of the following upgrade criteria in assessing the severity of psoriasis: involvement of visible areas, involvement of major parts of the scalp, involvement of genitals, involvement of palms and/or soles, onycholysis or onychodystrophy of at least two fingernails, pruritus leading to scratching, and the presence of recalcitrant plaques. 

### 3.4. Focus on Comorbidities

Prior to initiating systemic therapies for psoriasis, health care providers should consider the existence of comorbidities, as they may impact the choice of medication, the tolerability, and the adverse effects. Certain systemic agents could increase the risk of hepatotoxicity (e.g., methotrexate), especially in overweight or obese patients, which is also not rare in children [16]. Interestingly, children tolerate methotrexate in general quite well compared to adults, and it is regularly used in combination with biologics such as TNF alpha inhibitors to prevent the formation of anti-drug antibodies [16,17].

Extensive research has focused on the comorbidities associated with psoriasis and their effects on the quality of life (QOL) of the child and the guardian. Children suffering from psoriasis have a higher prevalence of obesity, diabetes mellitus, hypertension, uveitis, Crohn’s disease (CD), and psychiatric disorders, e.g., depressive disorders [1,3,18,19].

Comorbidities occur already in childhood, and thorough examinations should be performed regarding frequent comorbidities such as arthritic symptoms. The average age for psoriatic arthritis is between age 7 and 10, and about every third to fifth child with psoriasis develops arthritis. The sex ratio is about 60:40 female–male children. About half of children with psoriatic arthritis present joint symptoms prior to skin lesions, depending on age, and about 10% of children present with the onset of psoriasis of the skin and joints simultaneously [20]. The interval between both entities can be 2 to 10 years. Patients with juvenile psoriatic arthritis seem to form two distinct populations [21]: younger children under age 5 show more small joint involvement and dactylitis, whereas patients in later childhood tend to have more enthesitis, axial involvement, and oligoarthritis [21]. The diagnosis is made with arthritis and the presence of psoriasis of the skin or arthritis and at least two of the following criteria: dactylitis, nail pits, and psoriasis in a first-degree relative, which is present in about 40% of the children affected. In particular, the presence of dactylitis is one of the minor criteria of Vancouver [22,23]. Children with psoriatic arthritis seem to show nail changes more often than children with psoriasis of the skin without joint involvement (50–80% versus 30%). The association with spondylarthritis, enthesitis, uveitis, non-bacterial osteomyelitis (NBO), or chronic-recurrent multifocal osteomyelitis (CRMO) has to be considered with 30% positive testing for antinuclear antibodies (ANAs) and 50% for Human Leukocyte Antigen B (HLA) B27.

### 3.5. Systemic Therapeutics for Juvenile Psoriasis

#### 3.5.1. Preamble

With ongoing research about the cytokine patterns and immunological characteristics of psoriasis, the development of targeted systemic therapies became possible, and in the last decade, we have experienced a variety of options for systemic psoriasis medications for adults targeting the inflammatory cascade with biological disease-modifying antirheumatic drugs (bDMARDs); TNF-α blockers; interleukin (IL)-17, IL-12/23, or -23 inhibitors; phosphodiesterase inhibitors; and recently Janus kinase inhibitors (JAK inhibitors) [24]. Unfortunately, most of the new therapeutics have no approval for use in children, and therefore, our arsenal of choices for therapy is growing but is still limited and demands thorough preparation and information about treatment choices. One of the key pieces of information that needs to be discussed with parents/guardians is the limited approved medications for juvenile psoriasis [25,26]. At present, the federal approvals on both sides of the ocean differ significantly (Table 1), and we refer our patients to our European approvals. Moreover, following recent guidelines, the treatment of juvenile psoriatic arthritis should generally start with non-steroidal anti-inflammatory drugs (NSAIDs), the infiltration of the joints (ICS), or methotrexate. With loss of efficacy or in cases of treatment failure, second-line treatment with bDMARDs or JAK inhibitors or targeted synthetic disease-modifying antirheumatic drugs (tsDMARDs) is recommended [27], and some therapeutics make a clear distinction between psoriatic arthritis and juvenile idiopathic arthritis, which needs to be discussed with the pediatric rheumatologist. 

#### 3.5.2. In-Label Use

Adalimumab, a human monoclonal anti-TNF antibody targeting and blocking TNF-α for subcutaneous administration, was approved for use in children for the first time in 2008 [33]. Current approvals for children cover pediatric rheumatology, with the treatment of active polyarticular juvenile idiopathic arthritis (including polyarticular psoriatic arthritis [28,30]) in patients aged 2 years and older who had an inadequate response to one or more disease-modifying antirheumatic drugs, pediatric gastroenterology, and dermatology. Extensive safety studies are available [34]. Adalimumab is now approved for the treatment of severe chronic plaque psoriasis in children and adolescents aged 4 years and older (EMA), who have responded inadequately to topical therapy and phototherapies or for whom these therapies are not appropriate [35]. Dosage regimens regard body weight as cut off limits. For a body weight up to 30 kg, a subcutaneous administration of 20 mg is given every two weeks. Once the body weight reaches 30 kg or more, subcutaneous doses of 40 mg of Adalimumab are administered. The first two doses are given weekly, followed by dosing every two weeks subcutaneously.

Etanercept is a human dimeric fusion protein that functions as a TNF-α inhibitor by competitively binding to TNF and preventing its activation of the inflammatory cascade. Etanercept is indicated for the treatment of chronic severe plaque psoriasis in children age 6 years and older (EMA; age 4 and older FDA) who have had an inadequate response to other systemic or light therapy or have not tolerated them [36,37]. For the treatment of psoriatic arthritis, etanercept is approved for use in adolescents from age 12 and older who had an inadequate response or intolerance to methotrexate treatment. The dosage of Etanercept for treatment of plaque psoriasis is administered based on body weight adjustment in children and adolescents (patients under 62.5 kg should be administered 0.8 mg/kg (up to a maximum of 50 mg per dose) subcutaneously weekly with possible splitting of the dose and administration of half of the dose twice weekly).

Ustekinumab, an IL-12/23 monoclonal antibody, is approved for the treatment of plaque psoriasis in children and adolescents 6 years of age and older [38,39,40]. The recommended dosages are based on body weight. If body weight at the time of dosing is <60 kg, the recommended ustekinumab single dose is 0.75 mg/kg, administered every 12 weeks with an initial second dose 4 weeks after the first administration. For a body weight at the time of dosing between 60 and 100 kg, the recommended ustekinumab dose is 45 mg. For a body weight at the time of dosing of >100 kg, the recommended ustekinumab dose is 90 mg. Currently, there is no approval for juvenile arthritis by the EMA, although it has been approved by the FDA.

From the group of IL-17 inhibitors, Ixekizumab was the first IL-17A antagonist approved in 2020 for the treatment of pediatric patients with moderate to severe plaque psoriasis [41,42,43]. For children and adolescents aged six years and older with a body weight between 25 and 50 kg, an initial dose of 80 mg of Ixekizumab subcutaneously is recommended. Subsequently, children and adolescents should receive a maintenance dose of 40 mg subcutaneously every four weeks. For individuals with a body weight exceeding 50 kg, an initial dose of 160 mg and a maintenance dose of 80 mg every four weeks are recommended. There is no approval of ixekizumab for juvenile psoriatic arthritis so far.

Ixekizumab was followed by Secukinumab in the same year (2020) [44,45,46,47] for pediatric patients 6 years and older with moderate to severe plaque psoriasis who are candidates for systemic therapy or phototherapy. Secukinumab reached extended approval for juvenile psoriatic arthritis for children 6 years and older by the EMA in 2022 [47]. The recommended dosage depends on body weight, and it is administered as a subcutaneous injection with loading doses in weeks 0, 1, 2, 3, and 4, followed by monthly maintenance doses. Children and adolescents weighing less than 50 kg are to receive 75 mg each as both the loading and maintenance doses. For those with a body weight of 50 kg or more, loading and maintenance doses of 150 mg are recommended. In the case of an inadequate response, doses may be increased to 300 mg for individuals with a body weight of 50 kg or more.

Recently, JAK inhibitors complemented the options for the systemic therapy of juvenile psoriatic arthritis, and although they did not receive approval for pediatric psoriasis of the skin, studies could show improvement of the skin inflammation as well [48].

Over the last two decades, biologic therapies have brought about significant enhancements in the treatment outcomes for psoriasis and psoriatic arthritis. However, some patients exhibit primary failure to initial biologic agents or experience a decline in effectiveness over time (secondary failure). Several studies have indicated a clear association between the existence of anti-drug antibodies and instances of primary failure, secondary failure, and hypersensitivity reactions. Incorporating methotrexate therapy (MTX) resulted in a 67% reduction in the risk of anti-drug antibody development, as indicated by some studies (risk ratio 0.33; 95% CI 0.21, 0.52) [17].

#### 3.5.3. Off-Label Use

MTX is a folic acid antagonist, and it has anti-inflammatory and (mild) immunosuppressive properties that inhibit the production of inflammatory cytokines such as tumor necrosis factor (TNF)-α, IL-6, and IL-8. MTX is approved for adult psoriasis and in the pediatric population for inflammatory bowel disease (IBD) and juvenile arthritis. MTX is recommended by national guidelines and is regularly used as off-label treatment in juvenile psoriasis of the skin. With the new approvals for biologics, it loses its significance as a first-line option for this indication. It is used simultaneously with TNF-α blockers to prevent the production of antibodies against the drug, and a split dosage into two oral applications weekly can increase the acceptance with reduced side effects such as nausea or cephalgia [27,49].

**Figure 1:**
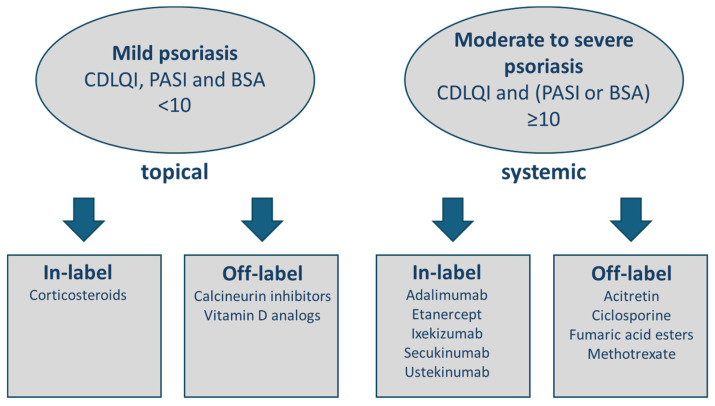
Therapy options for juvenile psoriasis of the skin. Overview of therapy options for the treatment of juvenile psoriasis of the skin in Europe, modified with reference to the German Guidelines S2K-Leitlinie “Therapie der Psoriasis bei Kindern und Jugendlichen AWMF Register Nr. 013-094, Update 2021” [24,25].

Further substances such as ciclosporine, fumaric acid esters, and retinoids do not have the approval for juvenile psoriasis of the skin and did not show advantages over modern approved biologics (Figure 1).

Other lower-ranking recommended systemic therapies for the treatment of psoriasis in children and adolescents in off-label use are apremilast, brodalumab, certolizumab pegol, guselkumab, and infliximab.

The information about this limited repertoire compared to systemic medications for adult patients must be discussed with the guardians to ensure a well-informed decision about treatment and to support compliance. This is significant for a treatment that has to be given over the long term to prevent destructive arthritis or a negative impact on the eyes or other organ systems from the inflammation.

### 3.6. Exchange with Pediatrician

**Figure 2:**
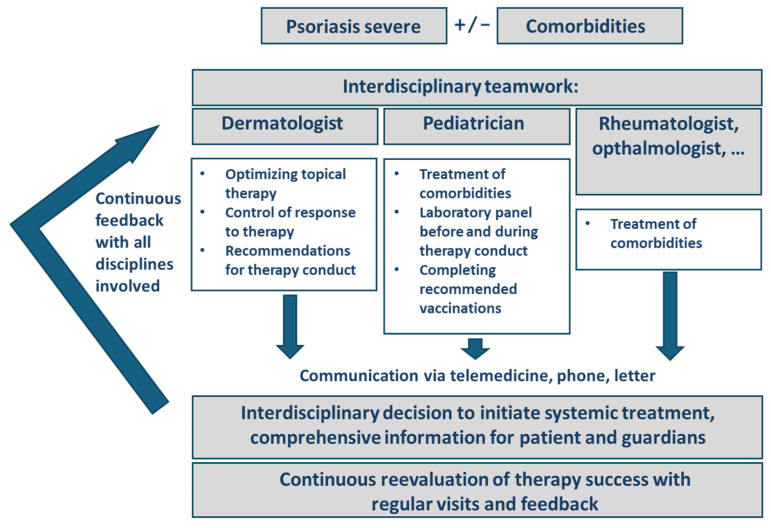
Interdisciplinary coworking before and during the initiation of systemic therapy for children with psoriasis.

For a comprehensive preparation of systemic therapies, a variety of explorations are mandatory before initiating and during the treatment with bDMARDs; thus, an interdisciplinary concept with other health care providers is helpful, especially for young patients. Figure 2 summarizes the explorations to be made before initiating treatment and the algorithm implemented in our management of pediatric cases. An interdisciplinary exchange with pediatricians helps to schedule the necessary check-ups and to address any problems that arise before and during therapy in the best possible way. Interdisciplinary exchange supports compliance with therapy, e.g., by explaining tips for dealing with side effects and how to proceed in the case of infections. The early implementation of a structured exchange and routine feedback of medical specialties facilitates disease management before ever starting any systemic therapy.

The cooperation with pediatricians helps to clarify pediatric specifics, e.g., for vaccinations and L/ATBI screening.

The application of biologics might require the preparation and teaching of guardians and pediatricians to enable them to take over, especially if the dosage depends on weight. The frequency of visits, laboratory controls, ophthalmologist check-ups, and travel distances should be addressed beforehand to assure the least interference with daily life, e.g., school attendance.

### 3.7. Laboratory Measures

Major changes in the full blood count and liver and kidney function impairment must be excluded before starting therapy, as well as the Tuberculosis interferon gamma release assay (TB-IGRA). The panel for initial laboratory testing in cases with arthritic involvement includes ANA, extractable nuclear antigen (ENA), lipase, serology for hepatitis and borreliosis, and HLA B27. During treatment, blood tests should be repeated regularly, normally four weeks after the initiation of systemic treatment and regularly in follow-ups every three months, but as infrequent as possible to minimize traumatic experiences for the children. In close consultation with pediatricians, blood samples can also be taken there if necessary.

### 3.8. Vaccination Status

Children with autoimmune or chronic inflammatory diseases and those undergoing immunomodulatory therapy have a higher risk of infection [50,51]. Consequently, a full vaccination status according to the national recommendations should be reviewed annually and any pending vaccinations applied. If possible, vaccinations should be administered during quiescent disease and 2–4 weeks prior to immunosuppressive treatment, but necessary treatment should never be postponed [52,53].

Inactivated vaccines can be applied at any time, and live-attenuated vaccines should be avoided during therapy with bDMARDs, except for the Measles, Mumps, and Rubella (MMR) booster or varicella-zoster virus (VZV) booster [51,52]. Thus, these live-attenuated vaccines are regarded as applicable only as boosters under systemic therapy with the biologics listed above for the treatment of children, although good communication between pediatricians and dermatologists is crucial for guardians to comprehend the recommendations if they vary from the approval status of the biologics mentioned above. The boosters for these live-attenuated vaccines are only recommended in cases of incomplete basic immunization following a strict protocol, published in a checklist for previous immunological testing before vaccinations [52,54]. Additional vaccination against SARS-CoV-2, influenza virus yearly, and the vaccine against pneumococcal infections is strongly recommended [55]. Constant changes in vaccination recommendations emphasize the importance of interdisciplinary exchange.

### 3.9. Tuberculosis Infection

Particularly noteworthy is the exclusion of latent tuberculosis infection (LTBI) or active tuberculosis infection (ATBI) prior to DMARD treatment.

Screening for L/ATBI is recommended, due to many cases of activation occurring during treatment with anti-TNF-α agents [25,56,57]. The issue has been discussed recently, because many bDMARDs are not interfering with tuberculostatic regulation, and the risk of infection or activation seems to be very low, particularly in low-incidence regions such as Western Europe [58,59]. Nevertheless, screening for L/ATBI is still mandatory before therapy with Etanercept, Adalimumab, and Ustekinumab but seems to be dispensable with Ixekizumab or secukinumab. Reference is made to national guidelines and the very useful heat map and consensus statement from 2022 [55]. The bDMARDs addressed in this handout are used in-label for children from 6 years of age; thus, screening procedures for tuberculosis refer to children of such age. An adjusted procedure is recommended for children < 5 years, according to the authors’ knowledge, clinical experience, and the national guideline [25,57,60].

Screening for LTBI prior to DMARDs includes Interferon-Gamma-Release Assay (IGRA) testing and, in some cases, chest X-Ray (one plane). In contrast to adults, children > 5 years should only receive a chest X-Ray if infection (by positive IGRA) is confirmed [25,57,60]. Some experts recommend performing chest X-Ray in adults only when patients are under immunosuppression prior to bDMARD therapy or the bDMARD planned is part of a higher-risk group, that is, Etanercept and Adalimumab, respectively [61]. Chest X-Rays, especially of younger children, should be preferably performed and evaluated by pediatric radiologists. If the results are inconclusive, pediatric pneumologists should be consulted without delay. If infection is confirmed and tuberculostatic therapy is required, patients should be referred to pediatric pneumologists trained in tuberculosis care for further treatment and controls.

LTBI is confirmed by positive IGRA and inconspicuous chest X-Ray [60] and is a non-infectious condition. Children can visit collective accommodations and communal facilities (e.g., schools). This information should be given to the parents/guardians.

ATBI is diagnosed by positive IGRA and specific infiltration in chest X-Ray or other organ manifestations [60]. Children with suspected or untreated ATBI should be classified as contagious, this must be clarified in hospital. Prompt treatment and isolation is of utmost importance. Local health authorities are informed immediately. In contrast to adults, children with ATBI are not contagious in general; however, the infectiousness increases with age. Infants and young children are almost never infectious for their environment. At primary school age, most children are rarely infectious, especially if they are not coughing. In adolescents, the infectivity is similar to that of adults [60].

The German national guideline suggests tuberculostatic therapy for at least 8 weeks prior to bDMARD initiation in children, irrespective of LTBI or ATBI [60,62]. In adults with LTBI, bDMARD therapy appears to be feasible after 4 weeks of tuberculostatic therapy, if the latter was free of adverse effects [61,63]. The initiation of bDMARDs during therapy for ATBI requires a good benefit–risk assessment [60,62,64].

In general, anti-agents should be avoided, if possible [57]. If problems occur during simultaneous medication, interdisciplinary exchange is suggested. There are recommendations available for adults [61]. It must be noted that most of the experience and quoted literature sources in this handout regarding tuberculosis and DMARD therapy refer to adults. Taking these things into consideration, and in expectation of future in-label use for younger children with psoriasis, the need for interdisciplinary exchange with a pediatrician increases.

## 4. Discussion

Pediatric therapy and management of psoriasis need special attention due to the different preconditions and approvals than for adults. The development and growth of a child cause constant adjustment to conditions. In expectation of future in-label use for younger children with psoriasis, the need for interdisciplinary exchange with a pediatrician increases.

Focusing on laboratory measures, preparations with vaccinations, and preliminaries for in-label and off-label therapeutics, it is obvious that a close interdisciplinary exchange is crucial for the success of systemic therapies in young patients. The interpretation and the consequences of laboratory and diagnostic results are too different from adult standards to exclude pediatricians from these decisions, especially in the choice of medication. Pediatric patients are as equally affected by comorbidities as adults, and therefore, a holistic management includes all disciplines involved. As an example, the management of TBI differs significantly from that in adults and needs to involve pediatric pneumologists. Figure 1 clearly demonstrates that the in-label and off-label decisions for medications in children are different as well, and we do not have the entire range of options as in adults (Table 1). Only 30% of the pediatric patients with psoriatic arthritis show a long-term drug-free remission. Therefore, a responsible preparation and thorough investigation of options for treatment are crucial in pediatric systemic therapy, especially if the child can benefit from approvals of medications for comorbidities with the same anti-inflammatory target. In our work with pediatric patients, we experienced an enormous benefit and time-saving process with close interdisciplinary communication, and therefore, we developed this algorithm (Figure 2) especially for lesser-experienced colleagues in gaining confidence in treatment decisions for young patients. The close coworking on special challenges with pediatric patients with psoriasis helps to implement early proper management of the disease and to avoid impaired development referring to age and social interaction.

## 5. Conclusions

Psoriasis is a common disease in children. As in adults, it is associated with significant comorbidity. Increased attention should be paid to the early detection and treatment of patients affected. With the increased options for the systemic treatment of children with psoriasis, clear and adapted information for the child, guardian, and pediatrician is essential to assure a well-managed environment and to prevent the unnecessary termination of effective therapy.

## Figures and Tables

**Table 1:** Overview of biologics (bDMARDs) and JAK inhibitors (tsDMARDs) approved for the treatment of juvenile psoriasis and for the treatment of juvenile psoriatic arthritis; note that some biologics received approval for treatment of juvenile idiopathic arthritis in general including psoriatic arthritis as a subcategory according to the Consensus Conference of the International League of Associations of Rheumatology (ILAR) in 2001 [28,29,30]. EMA, European Medicines Agency; FDA, Food and Drug Administration (from February 2024).

	Approval for Juvenile Severe ** Psoriasis of the Skin (A) In Europe (EMA) (B) In the U.S. (FDA)	Approval for Polyarticular Juvenile Idiopathic Arthritis or Psoriatic Arthritis * (A) In Europe (EMA) (B) In the U.S. (FDA)
Biologics (bDMARDs)		
Adalimumab	A First line: age 4 or older (April 2015) B No approval for this indication	A Second line: age 2 or older (February 2013) B First line: age 2 or older (since February 2008, changed in 2015 from age 4 to age 2)
Etanercept	A Second line: age 6 or older (August 2011) B First line: age 4 or older (November 2016)	A Second line: age 12 or older (July 2012) for psoriatic arthritis, age 2 and above for polyarticular juvenile idiopathic arthritis B Second line: age 2 or older (May 1999)
Ustekinumab	A Second line: age 6 or older (January 2020) B First line: age 6 and older (July 2020)	A No approval for this indication B Second line: age 6 or older (August 2022)
Ixekizumab	A First line: age 6 or older (June 2020) B First line: age 6 or older (March 2020)	A No approval for this indication B No approval for this indication
Secukinumab	A First line: age 6 or older (August 2020) B First line: age 6 or older (June 2021)	A Second line: age 6 or older (June 2022) B First line: age 2 or older (December 2021)
JAK inhibitors (tsDMARDs)		
Tofacitinib	A No approval for this indication B No approval for this indication	A Second line: age 2 or older B Second line: age 2 or older (December 2021)
Baricitinib	A No approval for this indication B No approval for this indication	A Second line: age 2 and older (July 2023) B No approval for this indication

* Some approvals such as for adalimumab in Europe have no explicit approval for psoriatic arthritis but for polyarticular juvenile idiopathic arthritis such as with psoriasis [31,32]. ** Adalimumab and Etanercept have approval only for severe forms of psoriasis in children; ustekinumab, secukinumab, and ixekizumab also for moderate to severe psoriasis.

## Data Availability

The data underlying this article will be shared upon reasonable request to the corresponding author.
